# Addition of a polygenic risk score, mammographic density, and endogenous hormones to existing breast cancer risk prediction models: A nested case–control study

**DOI:** 10.1371/journal.pmed.1002644

**Published:** 2018-09-04

**Authors:** Xuehong Zhang, Megan Rice, Shelley S. Tworoger, Bernard A. Rosner, A. Heather Eliassen, Rulla M. Tamimi, Amit D. Joshi, Sara Lindstrom, Jing Qian, Graham A. Colditz, Walter C. Willett, Peter Kraft, Susan E. Hankinson

**Affiliations:** 1 Channing Division of Network Medicine, Department of Medicine, Brigham and Women’s Hospital and Harvard Medical School, Boston, Massachusetts, United States of America; 2 Clinical and Translational Epidemiology Unit, Department of Medicine, Massachusetts General Hospital, Boston, Massachusetts, United States of America; 3 Department of Epidemiology, Harvard T.H. Chan School of Public Health, Boston, Massachusetts, United States of America; 4 Department of Cancer Epidemiology, H. Lee Moffitt Cancer Center and Research Institute, Tampa, Florida, United States of America; 5 Department of Biostatistics, Harvard T.H. Chan School of Public Health, Boston, Massachusetts, United States of America; 6 Department of Epidemiology, University of Washington, Seattle, Washington, United States of America; 7 Department of Biostatistics and Epidemiology, School of Public Health and Health Sciences, University of Massachusetts, Amherst, Massachusetts, United States of America; 8 Department of Surgery, Washington University School of Medicine, St. Louis, Missouri, United States of America; 9 Department of Nutrition, Harvard T.H. Chan School of Public Health, Boston, Massachusetts, United States of America; Vanderbilt University School of Medicine, UNITED STATES

## Abstract

**Background:**

No prior study to our knowledge has examined the joint contribution of a polygenic risk score (PRS), mammographic density (MD), and postmenopausal endogenous hormone levels—all well-confirmed risk factors for invasive breast cancer—to existing breast cancer risk prediction models.

**Methods and findings:**

We conducted a nested case–control study within the prospective Nurses’ Health Study and Nurses’ Health Study II including 4,006 cases and 7,874 controls ages 34–70 years up to 1 June 2010. We added a breast cancer PRS using 67 single nucleotide polymorphisms, MD, and circulating testosterone, estrone sulfate, and prolactin levels to existing risk models. We calculated area under the curve (AUC), controlling for age and stratified by menopausal status, for the 5-year absolute risk of invasive breast cancer. We estimated the population distribution of 5-year predicted risks for models with and without biomarkers. For the Gail model, the AUC improved (*p*-values < 0.001) from 55.9 to 64.1 (8.2 units) in premenopausal women (Gail + PRS + MD), from 55.5 to 66.0 (10.5 units) in postmenopausal women not using hormone therapy (HT) (Gail + PRS + MD + all hormones), and from 58.0 to 64.9 (6.9 units) in postmenopausal women using HT (Gail + PRS + MD + prolactin). For the Rosner–Colditz model, the corresponding AUCs improved (*p*-values < 0.001) by 5.7, 6.2, and 6.5 units. For estrogen-receptor-positive tumors, among postmenopausal women not using HT, the AUCs improved (*p*-values < 0.001) by 14.3 units for the Gail model and 7.3 units for the Rosner–Colditz model. Additionally, the percentage of 50-year-old women predicted to be at more than twice 5-year average risk (≥2.27%) was 0.2% for the Gail model alone and 6.6% for the Gail + PRS + MD + all hormones model. Limitations of our study included the limited racial/ethnic diversity of our cohort, and that general population exposure distributions were unavailable for some risk factors.

**Conclusions:**

In this study, the addition of PRS, MD, and endogenous hormones substantially improved existing breast cancer risk prediction models. Further studies will be needed to confirm these findings and to determine whether improved risk prediction models have practical value in identifying women at higher risk who would most benefit from chemoprevention, screening, and other risk-reducing strategies.

## Abstract 2

In a case-control study, Xuehong Zhang and colleagues study incorporation of genetic and other information into breast cancer risk models.

## Author summary

**Why was this study done?:**

- A polygenic risk score (PRS), mammographic density (MD), and postmenopausal endogenous hormone levels are all well-confirmed breast cancer risk factors.
- No prior study to our knowledge has comprehensively examined the joint contribution of these biological factors to improving existing breast cancer risk prediction models (i.e., the Gail model and the Rosner–Colditz model).

**What did the researchers do and find?:**

- We conducted a nested case–control study within the prospective Nurses’ Health Study and Nurses’ Health Study II that included 4,006 cases and 7,874 controls ages 34–70 years.
- We calculated the area under the curve (AUC), controlling for age and stratified by menopausal status, for the 5-year absolute risk of invasive breast cancer.
- We found that for both the Gail and Rosner–Colditz models, the AUC was significantly improved for both invasive and estrogen-receptor-positive breast cancers in postmenopausal women not using hormone therapy.
- Additionally, the percentage of 50-year-old women predicted to be at more than twice 5-year average risk (≥2.27%) was 0.2% for the Gail model alone and 6.6% for the Gail + PRS + MD + all hormones model.

**What do these findings mean?:**

- In this study, the addition of PRS, MD, and endogenous hormones substantially improved existing breast cancer risk prediction models.
- If confirmed, these findings may help identify women at higher risk who would most benefit from chemoprevention or other risk-reducing strategies.

## Introduction

Breast cancer is the most commonly diagnosed cancer in women. Risk prediction models have been developed to estimate an individual woman’s breast cancer risk, and have been used to both set clinical trial entry criteria [1] and provide tailored recommendations for screening, chemoprevention, and other risk-reducing strategies. Among such models, the Gail and Rosner–Colditz models have been well validated and have been used to identify high-risk women [2–4]. The original Gail model includes 5 confirmed risk factors (e.g., family history of breast cancer and reproductive factors) [3]; the Rosner–Colditz model includes the same factors plus additional established breast cancer risk factors such as BMI, alcohol intake, and postmenopausal hormone therapy (HT) use [5]. Both models are well calibrated in white populations, although their discriminatory ability is relatively modest [6,7]. Neither model includes biological markers of risk.

Multiple common genetic risk variants [8] and breast density [9], as measured on a mammogram, are additional well-confirmed breast cancer risk factors. Recent studies, though limited, have shown that including genetic risk variants (either individually or as a polygenic risk score [PRS]) and/or mammographic density (MD) significantly improves both the Gail model [10–17] and the Rosner–Colditz model [18]. In addition, considerable evidence supports an association of circulating estrogens, androgens, and prolactin with postmenopausal breast cancer risk [19–23]. These circulating hormones are only modestly correlated with either breast cancer genetic risk variants or MD [9]. We [24] and others [25] have recently found that incorporating hormones can improve breast cancer risk prediction. However, except for 1 small study on estrogen-receptor-positive (ER+) breast cancer [26], no other study to our knowledge has examined the degree to which incorporating all of these biological markers of risk improves risk prediction. Hence, we conducted an evaluation of the independent and joint contribution of PRS, MD, and endogenous hormone levels to the Gail and Rosner–Colditz models in the Nurses’ Health Study (NHS; follow-up 1976–2010) [27] and the Nurses’ Health Study II (NHSII; 1997–2009) [21].

## Methods

### Study population

The NHS was established in 1976 and included 121,700 female registered nurses aged 30–55 years [27], and the NHSII was established in 1989 and included 116,429 female registered nurses aged 25–42 years [21]. Questionnaires were mailed to women biennially to collect information on breast cancer risk factors, including age at menarche, age at first birth, parity, family history of breast cancer, height, weight, physical activity, menopausal status, age at menopause, and HT use. Alcohol consumption was assessed using a validated semi-quantitative food frequency questionnaire. This study was approved by the ethical review committees at Brigham and Women’s Hospital and the Harvard T.H. Chan School of Public Health. The completion and mailed return of the self-administered questionnaire was considered to imply informed consent of the participants.

### Outcome

We identified incident breast cancer cases up to 1 June 2010 through biennial questionnaires, confirmed the diagnoses with the participants (or next of kin), and obtained permission to collect relevant medical or pathology reports. We restricted this analysis to invasive breast cancer. We used tumor tissue microarrays (TMAs) [28,29] as the primary source to determine estrogen receptor (ER) status; when TMA data were not available (62%), we used medical/pathology reports to determine ER status. We observed high concordance (92%) of ER status between TMAs and medical/pathology reports [29,30].

### Gail and Rosner–Colditz models

In each cohort, we assessed 5-year breast cancer risk using the Gail and Rosner–Colditz risk scores. The Gail score includes number of first-degree relatives with a history of breast cancer, age at menarche, age at first birth, number of previous breast biopsies by age, and information on atypical hyperplasia at biopsy [2,3]. We used a modified Gail score [7] with family history (yes/no) and excluded data on atypical hyperplasia because it was not available. Because of its low prevalence, exclusion of atypical hyperplasia does not substantially alter the calibration in our cohorts [7].

The original factors included in the Rosner–Colditz model were age at menarche, age at first birth, birth index (a combination of number of children and birth spacing), family history of breast cancer, history of benign breast disease, premenopausal duration, type of menopause, postmenopausal duration, duration of postmenopausal HT use by type and timing, BMI, height, and alcohol intake [5]. Recently, we updated the Rosner–Colditz model by further including early life somatotype [18,31,32].

### Nested case–control study

The biospecimen collections for each cohort have been described in detail elsewhere [27,33–36]. Briefly, in the NHS, 32,826 participants provided blood samples in 1989–1990 and 18,743 women provided a second blood sample in 2000–2002. Among women who had not provided a blood sample, 29,684 provided a buccal cell sample in 2000–2006. In the NHSII, 29,611 participants provided blood samples in 1996–1999 and an additional 29,859 women provided cheek cells in 2004–2006. We conducted a nested case–control study among women who provided blood or buccal cell samples. We used risk set sampling and chose controls who were free from breast cancer at the same time point as the index case was diagnosed. In each cohort, we matched 1–2 control individuals by age (±1 year) and by month (±1) of biospecimen collection, and, for blood samples, by time of day (±2 hours), fasting status (≥10 hours since a meal versus <10 hours or unknown), menopausal status, and HT use at blood collection. Of note, we matched 2 controls for each postmenopausal case not using HT at blood collection and 1 control otherwise. For this study, we included invasive cases and matched controls and further included controls previously matched to in situ cases (*n* = 1,309) to improve statistical power. No women were identified more than once in this study. Sixty-one women initially identified as controls who subsequently became breast cancer cases were excluded from the analysis.

### PRS, MD, and laboratory assays

As previously reported [37], we used the TaqMan OpenArray SNP genotyping platform (Biotrove, Woburn, MA) to genotype 67 breast-cancer-associated variants identified from a large meta-analysis of 9 genome-wide association studies [8]. We created a PRS assuming a multiplicative joint effects model, and weighted SNPs by the logarithm of the odds ratio observed in the meta-analysis (S1 Table) [8]. Among women who provided blood samples in each cohort, we collected mammograms (pre-diagnostic for cases) and measured MD (i.e., percentage dense area, the dense area divided by the total area) in the NHS for breast cancer cases diagnosed through 2004 and matched controls, and in the NHSII through 2009 [9,38]. We previously measured testosterone (T), estrone sulfate (E1S), and prolactin (PRL) in pre-diagnostic plasma samples [19,39].

### Statistical analyses

We used unconditional logistic regression models to assess the relative risks (95% CIs) of invasive breast cancer with both continuous measures and quartile categories (based on control distributions) of PRS, MD, and circulating hormone levels, adjusting for matching factors. To evaluate improvement in risk prediction, we calculated the age-adjusted area under the curve (AUC) [40] as a measure of discrimination. Specifically, we compared the model including only a term for either the Gail or Rosner–Colditz risk score with a model with the risk score plus continuous measures of PRS, MD, and/or circulating hormones (each of the coefficients was obtained from age-adjusted logistic regression models). We computed separate age-specific AUCs and conducted a meta-analysis of these [40] to remove the effect of age. The population used in the analysis is shown in S2 Table. We have multiplied the AUC values by 100 for ease of presentation.

Since endogenous hormone–breast cancer associations differ by menopausal status and HT use [36], we evaluated the addition of plasma hormones only in the relevant subgroups. Specifically, for postmenopausal women not using HT at blood draw, we assessed the added value of T, E1S, and PRL. For postmenopausal women using HT at blood draw, we assessed the added value of PRL. We did not consider the addition of plasma hormones among premenopausal women because we observed weak or null associations in prior analyses [21,36]. MD and hormone levels were only available in a subset of women with genetic and questionnaire data (S2 Table). Thus, we imputed missing values to maintain the largest sample size possible. As previously described [41], we used linear regression to impute the values based on the beta coefficients obtained from a regression model, and added error to the predicted values; we used log transformed measured values as the outcome, and covariate predictors (including case status) selected based on previously published data and associations observed in our datasets. Details on imputing missing values are provided in S1 Methods.

Besides primary analyses stratified by menopausal status and HT use, we further evaluated the overall contribution of adding PRS, MD, and hormones among all invasive cases. Because the PRS and hormones are particularly strongly associated with ER+ breast cancer [8,39], we also repeated our analyses for ER+ tumors in postmenopausal women not using HT. For all models, we conducted sensitivity analyses excluding women with imputed values.

For the Gail model among postmenopausal women not using HT (the subset for which all the hormones, PRS, and MD were added), we also calculated the population distribution of predicted 5-year risks. To do this, we used the following data: (1) the relative risks from the nested case–control study [42,43], (2) the distribution of risk factors observed in our controls, and (3) age-specific incidence rates. The age-specific incidence rates were based on SEER (Surveillance, Epidemiology, and End Results; http://seer.cancer.gov/) data in white women for the years 2000–2008 (SEER17). We decided a priori to not conduct a similar analysis for the Rosner–Colditz model. Because we used the exposure distribution in our controls to estimate the population distribution, the greater the number of risk factors involved (e.g., BMI), the greater the chance our controls would not provide a sufficiently accurate reflection of the population distribution.

To assess potential model over-fitting, we used 10-fold cross-validation [24]. Lastly, we calculated the net reclassification index (NRI) to summarize the difference in proportion of individuals moving up in risk category minus the proportion moving down for those with breast cancer, and the proportion of individuals moving down minus the proportion moving up for those without breast cancer. NRI [44] is another measurement to evaluate the comparative discrimination ability of risk prediction models, and positive values of NRI indicate models’ improvement. For these analyses, we used twice the 5-year average risk (2.27%) in the general population to define the moving up or down groups (i.e., ≥2.27% versus <2.27%). We conducted all analyses using SAS version 9.2 (SAS Institute, Cary, NC). All statistical tests were 2-sided with a *p*-value < 0.05 indicating statistical significance. The analyses employed were all planned a priori. Additional study methods are provided in S1 Methods.

## Results

Compared with controls, cases were more likely to have benign breast disease, have breast cancer family history, and consume alcohol. Also, the PRS, MD, and circulating hormone levels were higher among cases than controls (Tables 1 and S3 for individuals included in the Gail and Rosner–Colditz models, respectively).

**Table 1:** Baseline characteristics of cases and controls in the Nurses’ Health Study (NHS; 1990) and Nurses’ Health Study II (NHSII; 1997) for the Gail model.

Characteristic	Cases	Controls
Number of cases/controls*	4,006	7,874
NHS	3,048	4,809
NHSII	958	3,065
**Demographic/lifestyle factors**		
**Age at blood draw, years, mean (SD)**	53.7 (8.0)	52.1 (8.3)
**Age at blood draw, years, *n* (%)**		
<40	139 (3%)	434 (6%)
40–44	415 (10%)	1,168 (15%)
45–49	928 (23%)	2,056 (26%)
50–54	769 (19%)	1,395 (18%)
55–59	711 (18%)	1,106 (14%)
60–64	635 (16%)	1,016 (13%)
65+	409 (10%)	699 (9%)
**BMI at blood draw, kg/m**^**2**^, **mean (SD)**	25.4 (4.0)	25.5 (4.5)
**Age at menarche, years, mean (SD)**	12.5 (1.4)	12.5 (1.4)
**Age at first birth, years, mean (SD)**	25.7 (3.7)	25.5 (3.7)
**Age at menopause, years, mean (SD)**	51.4 (4.3)	51.1 (5.2)
**Physical activity, MET-h/wk, mean (SD)**	16.3 (19.0)	17.0 (19.8)
**Alcohol consumption, g/d, mean (SD)**	4.8 (9.1)	4.2 (8.0)
**Parous, percent**	92%	94%
**Previous history of benign breast disease, percent**	50%	42%
**Family history of breast cancer, percent**	17%	11%
**Postmenopausal, percent**	61%	55%
**Postmenopausal women not using HT, percent**	25%	26%
**Postmenopausal women using HT, percent**	36%	29%
**Polygenic risk score**^†^, **mean**	0.19	−0.15
**Percent mammographic density, mean**	33.3	28.7
**5-year breast cancer risk score, mean**		
**Gail score, mean**	0.017	0.014
**Hormones, median (10–90th percentile)**		
Estrone sulfate (pmol/l)^‡^	723 (298, 1,772)	562 (240, 1,316)
Testosterone (nmol/l)^‡^	0.73 (0.40, 1.42)	0.66 (0.34, 1.26)
Prolactin (μg/l)^§^	10.6 (5.8, 19.3)	9.7 (5.5, 18.3)

*Cheek cell data were used for 3,411 women (1,369 cases/2,042 controls) in the NHS and 2,269 women (519 cases/1,750 controls) in the NHS II. Although age and menopausal status were matching factors, the addition of unmatched controls from the excluded in situ cases resulted in imbalance on age and menopausal status.

^†^The polygenic risk score was created using 67 independent SNPs previously identified in genome-wide association studies; the SNPs were weighted by the natural logarithm of their respective effect sizes and standardized (converted to mean = 0 and SD = 1) among all participants. A negative value indicates that a woman is less likely to develop breast cancer than the average-risk women, based on genetic data from these SNPs. Results presented in this table were among women who contributed to Gail model analyses.

^‡^Among postmenopausal women not using postmenopausal HT (1,005 cases/2,070 controls).

^§^Among postmenopausal women (2,435 cases/4,349 controls).

HT, hormone therapy; MET, metabolic equivalent task.

Each of the PRS, MD, and plasma hormones were significantly associated with invasive breast cancer risk. Similar results were observed when including imputed hormone and MD data (S4 and S5 Tables).

The AUC was statistically significantly improved with the combination of the PRS, MD, and hormones. For example, in postmenopausal women not using HT, where we included all these markers, the AUC improved from 55.5 (Gail) to 66.0 (Gail + PRS + MD + all hormones) for the Gail model (*p*-value < 0.001; Table 2). The AUC improved from 61.1 (Rosner–Colditz) to 67.4 (Rosner–Colditz + PRS + MD + all hormones) for the Rosner–Colditz model (*p*-value < 0.001; Table 3). The NRI values were 0.080 (95% CI 0.052–0.107) for the Gail model and 0.095 (95% CI 0.051–0.139) for the Rosner–Colditz model, indicating a significant improvement in the models’ discrimination ability. Significant, but more modest, improvements were observed for premenopausal women and for postmenopausal women using HT.

**Table 2:** Change in age-adjusted AUC of Gail model for invasive breast cancer by including PRS, percent MD, and circulating hormones.

Population and model	Number of cases/controls	AUC (95% CI)	Change in AUC (95% CI)
**Premenopausal women**	1,571/3,525	55.9 (54.1–57.7)*	
+ PRS		60.8 (59.0–62.6)	4.8 (2.8–6.8)
+ MD		61.1 (59.3–62.9)	5.2 (3.2–7.2)
+ PRS + MD		64.1 (62.5–65.7)	8.2 (6.0–10.4)
**Postmenopausal women not using HT**	1,005/2,070	55.5 (53.3–57.7)*	
+ PRS		61.1 (58.9–63.3)	5.8 (3.3–8.3)
+ MD		58.2 (56.0–60.4)	2.8 (0.6–5.0)
+ T + E1S + PRL		62.5 (60.3–64.7)	6.8 (4.3–9.3)
+ PRS + MD		62.4 (60.2–64.6)	7.0 (4.5–9.5)
+ PRS + T + E1S + PRL		64.8 (62.8–66.8)	9.4 (6.9–11.9)
+ MD + T + E1S + PRL		63.7 (61.5–65.9)	8.2 (5.7–10.7)
+ PRS + MD + T + E1S + PRL		66.0 (64.0–68.0)	10.5 (8.0–13.0)
**Postmenopausal women using HT**	1,430/2,279	58.0 (56.0–60.0)*	
+ PRS		61.9 (60.1–63.7)	3.9 (1.7–6.1)
+ MD		60.9 (58.9–62.9)	3.0 (0.8–5.2)
+ PRL		59.3 (57.3–61.3)	1.2 (-0.2–2.6)
+ PRS + MD		64.3 (62.5–66.1)	6.3 (4.1–8.5)
+ PRS + PRL		62.8 (61.0–64.6)	4.8 (2.6–7.0)
+ MD + PRL		62.0 (60.2–63.8)	3.9 (1.7–6.1)
+ PRS + MD + PRL		64.9 (63.1–66.7)	6.9 (4.7–9.1)
**All women**	4,006/7,874	56.3 (55.1–57.5)*	
+ PRS + MD + T + E1S + PRL		65.0 (64.0–66.0)	8.8 (7.4–10.2)

PRS, MD, and circulating hormones were modeled as continuous variables. Some of the changes in AUC did not match exactly to the difference in AUC shown in the table due to rounding.

*These are “baseline” AUC of the Gail model without any of the biomarkers included.

AUC, area under the curve; E1S, estrone sulfate; HT, hormone therapy; MD, mammographic density; PRL, prolactin; PRS, polygenic risk score; T, testosterone.

**Table 3:** Change in age-adjusted AUC of Rosner–Colditz model for invasive breast cancer by including PRS, percent MD, and circulating hormones.

Population and model	Number of cases/controls	AUC (95% CI)	Change in AUC (95% CI)
**Premenopausal women**	1,164/2,741	60.5 (58.5–62.5)*	
+ PRS		64.1 (62.1–66.1)	3.6 (2.0–5.2)
+ MD		63.3 (61.3–65.3)	2.7 (1.1–4.3)
+ PRS + MD		66.3 (64.5–68.1)	5.7 (3.9–7.5)
**Postmenopausal women not using HT**	653/1,336	61.1 (58.6–63.6)*	
+ PRS		64.9 (62.4–67.4)	3.8 (1.8–5.8)
+ MD		61.9 (59.4–64.4)	1.0 (-0.4–2.4)
+ T + E1S + PRL		64.3 (61.8–66.8)	3.1 (1.1–5.1)
+ PRS + MD		65.4 (62.9–67.9)	4.4 (2.2–6.6)
+ PRS + T + E1S + PRL		66.5 (64.0–69.0)	5.3 (2.9–7.7)
+ MD + T + E1S + PRL		65.2 (62.7–67.7)	4.0 (1.8–6.2)
+ PRS + MD + T + E1S + PRL		67.4 (64.9–69.9)	6.2 (3.8–8.6)
**Postmenopausal women using HT**	859/1,407	63.3 (60.9–65.7)*	
+ PRS		67.1 (64.7–69.5)	3.9 (1.9–5.9)
+ MD		65.9 (63.5–68.3)	2.4 (1.0–3.8)
+ PRL		64.6 (62.2–67.0)	1.1 (0.1–2.1)
+ PRS + MD		69.5 (67.3–71.7)	6.1 (3.9–8.3)
+ PRS + PRL		67.8 (65.4–70.2)	4.5 (2.3–6.7)
+ MD + PRL		66.7 (64.3–69.1)	3.1 (1.5–4.7)
+ PRS + MD + PRL		69.9 (67.7–72.1)	6.5 (4.3–8.7)
**All women**	2,676/5,484	61.8 (60.4–63.2)*	
+ PRS + MD + T + E1S + PRL		67.8 (66.6–69.0)	6.1 (4.9–7.3)

PRS, MD, and circulating hormones were modeled as continuous variables. Some of the changes in AUC did not match exactly to the difference in AUC shown in the table due to rounding.

*These are “baseline” AUC for the Rosner–Colditz model without any of the biomarkers included.

AUC, area under the curve; E1S, estrone sulfate; HT, hormone therapy; MD, mammographic density; PRL, prolactin; PRS, polygenic risk score; T, testosterone.

In sensitivity analyses, results were essentially the same when we excluded controls that were matched to in situ cases. Age-stratified results are shown in S6 and S7 Tables. We observed similar significant, albeit slightly stronger, improvements in risk prediction if we restricted our analyses to those women with measured MD and plasma hormone data (S8 and S9 Tables). In addition, for the Gail model, the average changes in age-adjusted AUC for 10-fold cross-validation among postmenopausal women not using HT were 10.4 for the 90% training dataset and 9.4 for the 10% validation dataset. The corresponding values were 6.2 and 5.9 for the Rosner–Colditz model. For ER+ tumors, the AUCs improved by 14.3 units for the Gail model and by 7.3 units for the Rosner–Colditz model (all *p*-values < 0.001) when including PRS, MD, and all hormones for postmenopausal women not using HT (S10 Table). We further provide the coefficients for each of the input parameters for our model (S11 Table).

**Fig 1:**
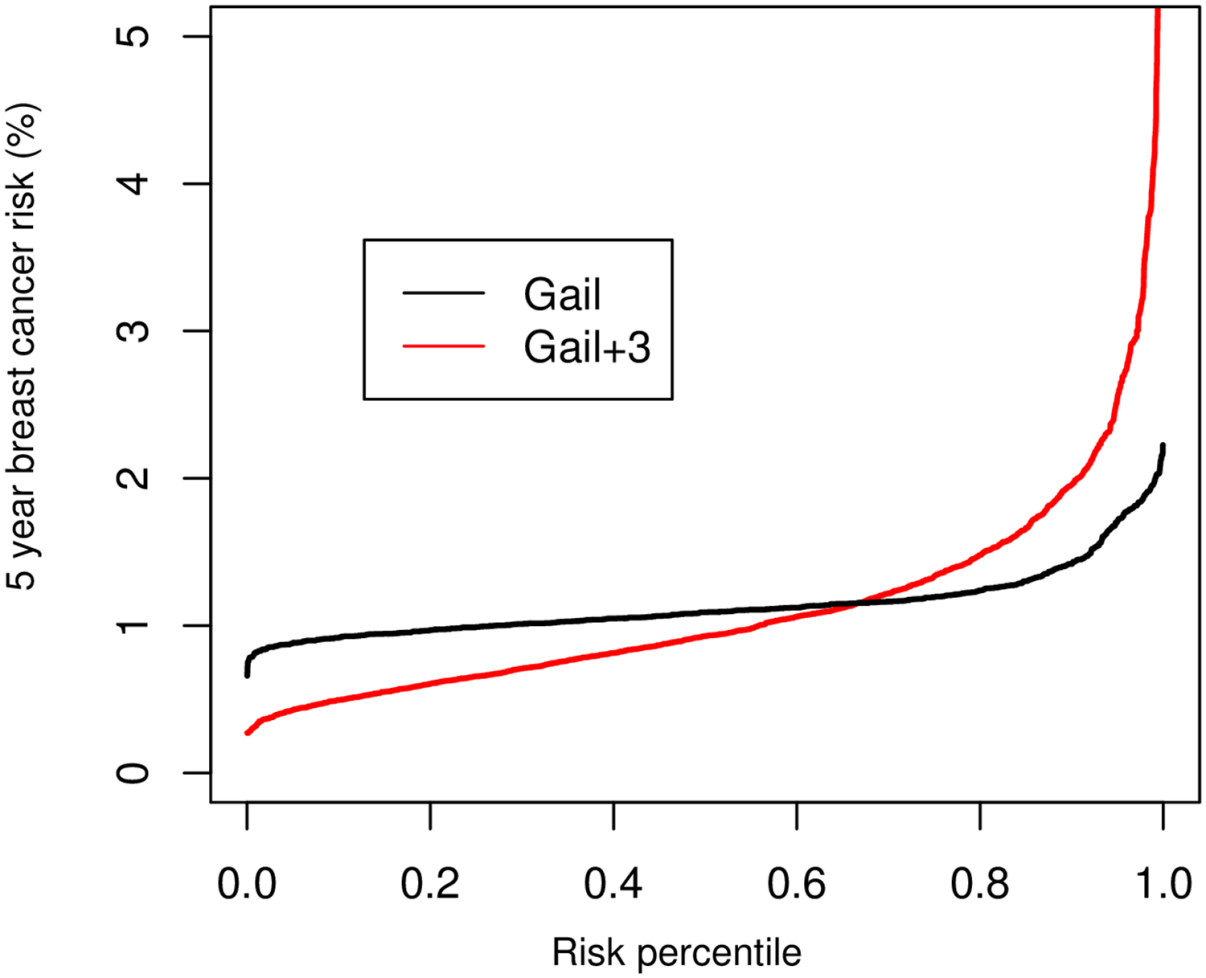
Predicted 5-year risk of breast cancer by risk score percentile comparing the Gail model to the Gail model plus 3 biological markers among postmenopausal women not using hormone therapy. In the figure, the *y*-axis was truncated at 5% because very few (0.69%) 50-year-old women were at 5-year risk at or greater than 5% to 12% (the maximum risk observed in our population) using the Gail + polygenic risk score + mammographic density + hormones (Gail + 3) model. The *x*-axis represents the risk percentile, ranging from >0 to 1 (i.e., a rank ordering of risk from >0% to 100% for the population).

For the Gail model among postmenopausal women not using HT, we calculated the distribution of 5-year absolute risk for 50-year-old women and found that including PRS, MD, and hormones better identified low- versus high-risk women (Fig 1). Specifically, the 5-year absolute risk (10th–90th percentile) ranged from 0.93% to 1.41% for Gail, and from 0.48% to 1.95% for Gail + PRS + MD + all hormones. Additionally, the Gail model alone predicted risk at or above 2.27% (i.e., twice average risk) for 0.2% of 50-year-old women, while the Gail + PRS + MD + all hormones model predicted risk at or above 2.27% for 6.6% of 50-year-old women.

## Discussion

To our knowledge, this is the first study to examine the added value of incorporating multiple biomarkers, including PRS, MD, and endogenous hormones, into the Gail and Rosner–Colditz models. Although these factors individually and together improved risk prediction for both invasive and ER+ breast cancer, the model improved the most among postmenopausal women not using HT, where each of the biomarkers significantly predicted risk.

Although only 1 prior study [26] has considered PRS, MD, and endogenous hormones together, our observed associations for including individual biomarkers were consistent with previous findings [9,45,46]. For example, in our study the observed added value of the PRS (improved AUC by 3.9–5.8 units) and MD (improved AUC by 2.8–5.2) to the Gail model was comparable to that in previous studies, with AUC increases of 3–7 units after adding 9 to 77 SNPs versus the Gail model alone [10,11,45,47–49] and of 2–5 units when including Breast Imaging Reporting and Data System (BI-RADS) categories or continuous MD [15,50,51]. Given their largely independent nature, we further found that any combination of the biomarkers improved prediction beyond the addition of a single biomarker.

Furthermore, among postmenopausal women not using HT, we were able to include 3 endogenous hormones—representing 3 hormone axes only modestly correlated—which improved the model by a similar magnitude as that observed for PRS and MD. Including all these factors together improved the model the most (i.e., by 10.5 units versus the Gail model alone). We selected a minimal set of hormones that have been shown to provide the largest prediction improvement for invasive breast cancer [24]. Circulating levels of these hormones are, at most, modestly correlated with either the individual breast cancer SNPs or MD [9,46]. The overall increase in AUC when adding all biomarkers was less for the Rosner–Colditz than the Gail model, likely because estrogens and MD are strongly associated with BMI, which is included in the former model. Our results are consistent with prior studies (including our own) that have suggested independent, potentially additive associations for PRS, MD, and hormones with breast cancer risk [9,46]. For example, compared with postmenopausal women in the lowest tertile of both MD and T, women at the highest tertile of both were at 5- to 6-fold greater risk of breast cancer.

Notably, in our study population, only approximately 45% of postmenopausal women were not using HT, as the blood collections occurred during a period of time when HT was used more frequently than in the US today. In contrast, based on 1999–2010 data from the US National Health and Nutrition Examination Survey (NHANES), about 95% of postmenopausal women are not current HT users [52]. This suggests that the predictive improvement provided by adding PRS, MD, and hormones in the US population as a whole will be larger than what we observed in this study. Furthermore, we found greater improvements in the AUC among ER+ tumors than among all invasive breast cancers, suggesting that development of a risk prediction model specifically for ER+ disease might lead to better discrimination ability and more targeted chemoprevention [53,54].

Multiple issues important to the potential clinical use of these biomarkers for risk prediction still need to be addressed. Our prior work found that circulating hormone levels may only need to be measured every 10 years [39], although confirmation is needed. Further, an assessment of the cost of including endogenous hormones and risk SNPs relative to the added predictive value needs to be considered. Currently, MD is clinically reported using the BI-RADS classification system; future study should evaluate whether using the computer-assisted thresholding method (used here) or other newly developed automated methods would add significantly more information for risk prediction. In addition, the Endocrine Society and the Centers for Disease Control and Prevention have recently developed a national standardization program for the measurement of T and estradiol [55,56], thus facilitating accurate measurement of these hormones on a routine basis, but E1S and PRL measurements still need to be addressed. Importantly, further assessments of clinical usefulness, including relative benefits and harms (both objective and subjective) of actions taken after receiving a risk score (e.g., chemoprevention) also are needed in future work.

Limitations of this study deserve comment. Although this study represents one of the largest to date to evaluate the contribution of measured MD or hormone levels to risk prediction, we did not have such data on all women. However, the baseline AUCs were similar among women with measured and imputed data, and results were similar when using measured data. Because of our nested case–control design with age matching, we were not able to assess the contribution of age to the AUC, and our absolute AUC values are as a result lower than those including age would be. Also, we only included 67 SNPs, although a larger set of SNPs (at least 94) has been identified [57], which may further improve discrimination. In calculating Gail model absolute risks (with and without biomarkers), we used the risk factor distribution in our controls to estimate the distribution in the general non-Hispanic white population, in part because population distributions were not available for the biomarkers. This may have caused either an over- or underestimation of our risk estimates. To minimize assumptions made about comparability between our controls and the general population (e.g., in BMI), we only evaluated absolute risk using the simpler Gail model. In addition, it is critical to assess model calibration, and to validate these models in independent populations, which was beyond the scope of the current work. However, there are several reasons to believe our findings will hold up. We utilized 2 well-validated statistical models as the base model, the exposures added to these models were each very well confirmed and quite well quantified breast cancer risk factors, and our cross-validation analyses suggested little to no over-fitting. Lastly, our study population was predominantly white: It will be important to identify the best subset of biomarkers for breast cancer, as well as evaluate their added value, in additional racial/ethnic populations [58,59].

In summary, our findings indicate that the incorporation of multiple biomarkers improves the current Gail and Rosner–Colditz models for both total invasive breast cancer and ER+ breast cancer. If validated in independent populations, our findings could help identify women at higher risk who would most benefit from chemoprevention, screening, and other risk-reducing strategies.

## Supporting information

S1 Methods (DOCX)

S1 STROBE Statement (DOCX)

S1 Table SNPs included in the PRS: Allele frequencies, odds ratios, and weights. (DOCX)

S2 Table The number of cases and controls with available biological marker data: NHS and NHSII. (DOCX)

S3 Table Baseline characteristics of cases and matched controls in the NHS (1990) and NHSII (1997) for the Rosner–Colditz model. (DOCX)

S4 Table Multivariable relative risk (MV RR) of invasive breast cancer in relation to PRS and MD in the Gail model. (DOCX)

S5 Table Multivariable relative risk (MV RR) of invasive breast cancer in relation to circulating pre-diagnostic hormones in the Gail model. (DOCX)

S6 Table Change in age-adjusted AUC of Gail model by age group for invasive breast cancer by including PRS, MD, and circulating hormones. (DOCX)

S7 Table Change in age-adjusted AUC of Rosner–Colditz model by age group for invasive breast cancer by including PRS, MD, and circulating hormones. (DOCX)

S8 Table Change in age-adjusted AUC of Gail model for invasive breast cancer by including PRS, measured MD, and measured circulating hormones. (DOCX)

S9 Table Change in age-adjusted AUC of Rosner–Colditz model for invasive breast cancer by including PRS, measured MD, and measured circulating hormones. (DOCX)

S10 Table Change in age-adjusted AUC of Gail and Rosner–Colditz models for ER+ breast cancer by including PRS, MD, and circulating hormones among postmenopausal women not using HT. (DOCX)

S11 Table Beta coefficients for each of the input parameters used in Tables 2 and 3. (DOCX)

## Abbreviations

AUC: area under the curve
BI-RADS: Breast Imaging Reporting and Data System
E1S: estrone sulfate
ER: estrogen receptor
ER+: estrogen-receptor-positive
HT: hormone therapy
MD: mammographic density
NHS: Nurses’ Health Study
NHSII: Nurses’ Health Study II
NRI: net reclassification index
PRL: prolactin
PRS: polygenic risk score
T: testosterone
