# Supplementary material for: Addition of a polygenic risk score, mammographic density, and endogenous hormones to existing breast cancer risk prediction models: A nested case–control study
Source: PLoS Med. 2018 Sep 4;15(9):e1002644. doi: 10.1371/journal.pmed.1002644 (PMC6122802; doi:10.1371/journal.pmed.1002644)
Supplement: S1 Methods — (DOCX) [file pmed.1002644.s001.docx]

**S1 Methods**

**Nested case-control study**

In NHS and NHSII, women who provided biospecimen samples were similar to the entire cohort with respect to major breast cancer risk factors such as age and BMI.^1^ Among participants who provided biospecimens, the follow-up rate was 97% in 2012 for NHS participants, and 88% in 2013 for NHSII participants.^2^

**Polygenic risk score (PRS)**

Our methods for genomic DNA extraction from blood samples and buccal cells have been described elsewhere.^3^ In brief, we used the TaqMan Open Array SNP genotyping platform (Biotrove, Woburn, MA) with 384-well format TaqMan assays to genotype 67 breast cancer associated variants identified from a meta-analysis of nine GWAS studies, including 10,052 breast cancer cases and 12,575 controls of European ancestry.^4^ TaqMan primers and probes were designed using Primer Express Oligo Design software v2.0 (ABI PRISM). Primers, probes, and conditions for genotyping assays are available upon request. Missing SNPs were imputed based on case-control status and, when available, SNPs in high linkage disequilibrium (R^2^ >0.8) with the SNP of interest. We created a PRS assuming a multiplicative joint effects model, and weighted by the logarithm of the effect sizes observed in the meta-analysis.^4^

**Mammographic density (MD)**

The collection of mammograms and measurement of MD in our cohorts have been described in detail elsewhere.^5,6^ In brief, among women who provided blood samples in each cohort, we collected mammograms (pre-diagnosis for cases) and measured MD (i.e., percentage dense area, the dense area divided by the total area) in the NHS for breast cancer cases diagnosed through 2004 and matched controls, and NHSII through 2009. Women with mammograms were similar to those for whom images were unattainable with respect to age, BMI, and circulating hormone levels.^5,6^ The craniocaudal views of both breasts were digitized with a Lumisys 85 digital laser film scanner (Lumisys, Sunnyvale, CA, USA) or VIDAR CAD PRO Advantage scanner (VIDAR Systems Corporation; Herndon, VA) using comparable resolution of 150 dots per inch and 12 bit depth. We used Cumulus software (Canto Software Inc, San Francisco, CA, USA) for computer-assisted thresholding to measure the percentage of dense area. The observer was blinded to the case-control and quality control status of the mammograms. For each batch, the within-person intraclass correlation coefficient has been at least 0.90.^6^ We used the average percent density of both breasts for this analysis because this variable has been shown as the strongest risk factor for breast cancer in this population.^6^

**Laboratory assays**

We previously measured testosterone (T), estrone sulfate (E1S), and prolactin (PRL) in pre-diagnostic plasma samples.^7,8^ T was assayed using an extraction step and column chromatrography followed by radioimmunoassay (RIA) (for cases diagnosed up to 2000) or liquid chromatography-tandem mass spectrometry (LC-MS/MS). For E1S, cases and controls identified through 2004 were assayed at the University of Massachusetts Medical Center Longcope Laboratory (Worcester, MA) or at Quest Diagnostics by radioimmunoassay. The remaining samples were assayed at the Mayo Clinic by LC-MS/MS after extraction. In pilot samples assayed by RIA and MS methods (n=10-21), the Pearson correlation was 0.96 (95%CI=0.90-0.98) for T and 0.98 (95%CI=0.93-0.99) for E1S.^9^ PRL was measured by microparticle enzyme immunoassay. Cases and controls and samples from the same woman over time were assayed together in the same run and the case-control sets were labeled to mask case-control status. The coefficient of variation (CVs) of blinded quality control samples was <10% for 67% of batches and 10% to 15% otherwise.^9^

**Statistical analyses**

Since endogenous hormone-breast cancer associations differ by menopausal status and HT use, ^10,11^ we evaluated the addition of plasma hormones only in the relevant subgroups. Specifically, for postmenopausal women not using HT at blood draw, we assessed the added value of T, E1S, and PRL, with or without PRS or MD. For postmenopausal women using HT at blood draw, we assessed the added value of PRL, with or without PRS or MD. For NHS cases and matched controls that occurred between 1990 and 2000 we used T, E1S, and PRL measured in 1990 (and other covariate information in 1990); for NHS cases and matched controls that occurred between 2000 and 2010, we used T and E1S measured in 1990, but PRL measured in 2000 (and other covariate information in 2000), because PRL levels are only associated with risk approximately within 10 years after blood draw,^8^ while the other hormones predict risk for up to 20 years.^7^ For NHSII cases and matched controls that occurred between 1997 and 2009 we used T and PRL measured in 1997 (and other covariate information in 1997). We did not consider the addition of plasma hormones among premenopausal women because we observed weak or null associations in prior analyses.^12^ We had complete genetic and questionnaire (i.e., traditional breast cancer risk factors) data on 11,880 women (4,006 cases/7,874 controls) for Gail model analyses and 8,160 women (2,676 cases/5,484 controls) for Rosner-Colditz model analyses. Fewer women contributed to the Rosner-Colditz model, because of missing data on several risk factors, particularly BMI at age 18 and early life somatotype [Rice et al].

MD and hormone levels were only available in a subset of women with genetic and questionnaire data. Thus, we imputed missing values to maintain the largest sample size possible. As previously described,^13^ we used linear regression to impute the values based on the beta-coefficients obtained from a regression model with measured values (log transformed to improve normality) as the outcome, and covariate predictors selected based on previously published data and associations observed in our data sets. Case status also was included as a predictor, so that the difference in hormone level (or MD) distribution between cases and controls observed in the subset with measured hormone levels (or measured MD) would be maintained in the larger data set. Normally distributed residual errors were added to the predicted values so that the distribution of imputed values would be similar to the observed value distribution. For MD, the predictors were: age, case status, adolescent somatotype, BMI, age at first birth, parity, and menopausal status.^14^ For E1S, the predictors were age, BMI and alcohol consumption at blood draw, and case status. For T, we included smoking, parity, hysterectomy, steroid use, and case status. For PRL, we included the predictors were time of blood draw, steroid use, and case status. We evaluated the association between each hormone and breast cancer risk by creating 5 imputed datasets. We used multiple imputation methods to combine estimates^15^ and found that the results were essentially the same comparing the first imputation with an average of five imputations (**S3 Table**). Additionally, we compared the results using singly imputed data vs. multiply imputed data with 50 imputations and found essentially the same results among postmenopausal women not using HT (the subset for which all the hormones, GRS and MD were added). Specifically, for the Gail model, the AUC changes (95%CIs) were 10.5 (8.0-13.0) for 1 imputation (Table 2) vs. 10.3 (7.3-13.2) for 50 imputations. For the Rosner-Colditz model, the AUC changes (95%CIs) were 6.2 (3.8-8.6) for 1 imputation (Table 3) vs. 6.6 (4.1-9.1) for 50 imputations. Hence, we used hormone values from the first imputation in the results presented. In addition to primary analyses stratified by menopausal status and HT use, we further evaluated the overall contribution of adding PRS, MD, and hormones among all women. We created a model that included indicators for menopausal status (premenopausal women, postmenopausal women using HT), and PRS, MD, and each of the hormones for all women if appropriate. Using a Wald test based on the unconditional logistic regression model, we tested the interaction between MD and menopausal status, as well as between PRL and menopausal status, to evaluate whether adding this interaction term improved the model, but it did not (all p-values>0.05).

**References**

1. Tworoger SS, Sluss P, Hankinson SE. Association between plasma prolactin concentrations and risk of breast cancer among predominately premenopausal women. Cancer Res 2006; **66**(4): 2476-82.

2. Poole EM, Schernhammer E, Mills L, Hankinson SE, Tworoger SS. Urinary melatonin and risk of ovarian cancer. Cancer Causes Control 2015; **26**(10): 1501-6.

3. Hiraki LT, Joshi AD, Ng K, et al. Joint effects of colorectal cancer susceptibility loci, circulating 25-hydroxyvitamin D and risk of colorectal cancer. PloS one 2014; **9**(3): e92212.

4. Michailidou K, Hall P, Gonzalez-Neira A, et al. Large-scale genotyping identifies 41 new loci associated with breast cancer risk. Nat Genet 2013; **45**(4): 353-61, 61e1-2.

5. Pettersson A, Hankinson SE, Willett WC, Lagiou P, Trichopoulos D, Tamimi RM. Nondense mammographic area and risk of breast cancer. Breast Cancer Res 2011; **13**(5): R100.

6. Tamimi RM, Byrne C, Colditz GA, Hankinson SE. Endogenous hormone levels, mammographic density, and subsequent risk of breast cancer in postmenopausal women. J Natl Cancer Inst 2007; **99**(15): 1178-87.

7. Zhang X, Tworoger SS, Eliassen AH, Hankinson SE. Postmenopausal plasma sex hormone levels and breast cancer risk over 20 years of follow-up. Breast Cancer Res Treat 2013; **137**(3): 883-92.

8. Tworoger SS, Eliassen AH, Zhang X, et al. A 20-year prospective study of plasma prolactin as a risk marker of breast cancer development. Cancer Res 2013; **73**(15): 4810-9.

9. Tworoger SS, Zhang X, Eliassen AH, et al. Inclusion of endogenous hormone levels in risk prediction models of postmenopausal breast cancer. J Clin Oncol 2014; **32**(28): 3111-7.

10. Hankinson SE, Eliassen AH. Endogenous estrogen, testosterone and progesterone levels in relation to breast cancer risk. J Steroid Biochem Mol Biol 2007; **106**(1-5): 24-30.

11. Hankinson SE, Willett WC, Manson JE, et al. Plasma sex steroid hormone levels and risk of breast cancer in postmenopausal women. J Natl Cancer Inst 1998; **90**(17): 1292-9.

12. Fortner RT, Eliassen AH, Spiegelman D, Willett WC, Barbieri RL, Hankinson SE. Premenopausal endogenous steroid hormones and breast cancer risk: results from the Nurses' Health Study II. Breast Cancer Res 2013; **15**(2): R19.

13. Rosner B, Colditz GA, Iglehart JD, Hankinson SE. Risk prediction models with incomplete data with application to prediction of estrogen receptor-positive breast cancer: prospective data from the Nurses' Health Study. Breast Cancer Res 2008; **10**(4): R55.

14. Rice MS, Tworoger SS, Hankinson SE, et al. Breast cancer risk prediction: an update to the Rosner-Colditz breast cancer incidence model. Breast Cancer Res Treat 2017; **166**(1): 227-40.

15. Rubin D. Multiple Imputation for Nonresponse in Surveys. New York: Wiley 1987.
