# Supplementary material for: Addition of a polygenic risk score, mammographic density, and endogenous hormones to existing breast cancer risk prediction models: A nested case–control study
Source: PLoS Med. 2018 Sep 4;15(9):e1002644. doi: 10.1371/journal.pmed.1002644 (PMC6122802; doi:10.1371/journal.pmed.1002644)
Supplement: S1 STROBE Statement — (DOCX) [file pmed.1002644.s002.docx]

STROBE statement checklist of items that should be included in reports of observational studies

|  | **Item No** | **Recommendation** |  |
| --- | --- | --- | --- |
| **Title and abstract** | | |  |
|  | 1 | (*a*) Indicate the study's design with a commonly used term in the title or the abstract | We mentioned prospective cohort design in the abstract |
|  |  | (*b*) Provide in the abstract an informative and balanced summary of what was done and what was found | We have provided an informative and balanced summary in the abstract. |
| **Introduction** | | |  |
| Background/rationale | 2 | Explain the scientific background and rationale for the investigation being reported | Introduction section, paragraphs 1, 2 |
| Objectives | 3 | State specific objectives, including any prespecified hypotheses | Introduction section, paragraphs 1, 2 |
| **Methods** | | |  |
| Study design | 4 | Present key elements of study design early in the paper | Methods section, |
| Setting | 5 | Describe the setting, locations, and relevant dates, including periods of recruitment, exposure, follow-up, and data collection | Methods section, paragraph 1 |
| Participants | 6 | (*a*) *Cohort study*?Give the eligibility criteria, and the sources and methods of selection of participants. Describe methods of follow-up*Case-control study*?Give the eligibility criteria, and the sources and methods of case ascertainment and control selection. Give the rationale for the choice of cases and controls*Cross sectional study*?Give the eligibility criteria, and the sources and methods of selection of participants | This is cohort study. We have provided the required information in methods section. |
|  |  | (*b*) *Cohort study*?For matched studies, give matching criteria and number of exposed and unexposed*Case-control study*?For matched studies, give matching criteria and the number of controls per case | We have mentioned relevant information in method section. |
| Variables | 7 | Clearly define all outcomes, exposures, predictors, potential confounders, and effect modifiers. Give diagnostic criteria, if applicable | Methods section, paragraphs 3,4,5 |
| Data sources/ measurement | 8* | For each variable of interest, give sources of data and details of methods of assessment (measurement). Describe comparability of assessment methods if there is more than one group | Methods section, paragraphs 4,5 |
| Bias | 9 | Describe any efforts to address potential sources of bias | Methods section. |
| Study size | 10 | Explain how the study size was arrived at | Methods section see study population. |
| Quantitative variables | 11 | Explain how quantitative variables were handled in the analyses. If applicable, describe which groupings were chosen and why |  |
| Statistical methods | 12 | (*a*) Describe all statistical methods, including those used to control for confounding | Statistical analysis section. |
|  |  | (*b*) Describe any methods used to examine subgroups and interactions | Statistical analysis section. |
|  |  | (*c*) Explain how missing data were addressed | Statistical analysis section. |
|  |  | (*d*) *Cohort study*?If applicable, explain how loss to follow-up was addressed*Case-control study*?If applicable, explain how matching of cases and controls was addressed*Cross sectional study*?If applicable, describe analytical methods taking account of sampling strategy | Methods section. |
|  |  | (*e*) Describe any sensitivity analyses | Statistical analysis section, paragraph 5 |
| **Results** | | |  |
| Participants | 13* | (*a*) Report numbers of individuals at each stage of study?eg numbers potentially eligible, examined for eligibility, confirmed eligible, included in the study, completing follow-up, and analysed | Methods section, paragraphs 1,2,4 |
|  |  | (*b*) Give reasons for non-participation at each stage | Methods section. |
|  |  | (*c*) Consider use of a flow diagram | We have not used diagram but the description should be enough for readers to understand how we got these numbers. |
| Descriptive data | 14* | (*a*)Give characteristics of study participants (eg demographic, clinical, social) and information on exposures and potential confounders | Results section, paragraph 1 |
|  |  | (*b*) Indicate number of participants with missing data for each variable of interest | Results section, paragraph 1 |
|  |  | (*c*) *Cohort study*?Summarise follow-up time (eg average and total amount) | Results section, paragraph 1 |
| Outcome data | 15* | *Cohort study*?Report numbers of outcome events or summary measures over time | Results section, paragraph 1 |
|  |  | *Case-control study?*Report numbers in each exposure category, or summary measures of exposure |  |
|  |  | *Cross sectional study?*Report numbers of outcome events or summary measures |  |
| Main results | 16 | (*a*) Report the numbers of individuals at each stage of the study?eg numbers potentially eligible, examined for eligibility, confirmed eligible, included in the study, completing follow-up, and analysed | Results section, paragraph 1 |
|  |  | (*b*) Give reasons for non-participation at each stage | Methods and Results sections. |
|  |  | (*c*) Consider use of a flow diagram | We have not used diagram but the description should be enough for readers to understand how we got these numbers. |
| Other analyses | 17 | Report other analyses done?eg analyses of subgroups and interactions, and sensitivity analyses | Results section, paragraph 3 |
| **Discussion** | | |  |
| Key results | 18 | Summarise key results with reference to study objectives | Discussion section, paragraph 1 |
| Limitations | 19 | Discuss limitations of the study, taking into account sources of potential bias or imprecision. Discuss both direction and magnitude of any potential bias | Discussion section, paragraph 6 |
| Interpretation | 20 | Give a cautious overall interpretation of results considering objectives, limitations, multiplicity of analyses, results from similar studies, and other relevant evidence | Discussion section, paragraphs 2, 3, 4 |
| Generalisability | 21 | Discuss the generalisability (external validity) of the study results | Discussion section, paragraph 6 |
| **Other information** | | |  |
| Funding | 22 | Give the source of funding and the role of the funders for the present study and, if applicable, for the original study on which the present article is based | Acknowledgement section. |

*Give information separately for cases and controls in case-control studies and, if applicable, for exposed and unexposed groups in cohort and cross sectional studies.

The STROBE checklist is best used in conjunction with the explanation and elaboration article.^18-20^ This article and separate versions of the checklist for cohort, case-control, and cross sectional studies are available at [www.strobe-statement.org](http://www.strobe-statement.org).
