# Supplementary material for: Addition of a polygenic risk score, mammographic density, and endogenous hormones to existing breast cancer risk prediction models: A nested case–control study
Source: PLoS Med. 2018 Sep 4;15(9):e1002644. doi: 10.1371/journal.pmed.1002644 (PMC6122802; doi:10.1371/journal.pmed.1002644)
Supplement: S1 Table — (DOCX) [file pmed.1002644.s003.docx]

**S1 Table. SNPs included in the polygenic risk score: Allele frequencies, odds ratios and weights.**

| SNP | Major | Minor | Risk allele | Risk allele frequency^1^ | OR^2^ | Risk allele frequency^3^ | Weight^4^ |
| --- | --- | --- | --- | --- | --- | --- | --- |
| rs10069690 | C | T | T | 0.26 | 1.06 | 0.26 | 0.058 |
| rs1011970 | G | T | T | 0.17 | 1.06 | 0.17 | 0.058 |
| rs1045485 | G | C | G | 0.87 | 0.97 | 0.88 | 0.030 |
| rs10472076 | T | C | C | 0.38 | 1.05 | 0.37 | 0.049 |
| rs10483813 | C | T | C | 0.77 | 0.92 | 0.77 | 0.083 |
| rs10771399 | A | G | A | 0.88 | 0.86 | 0.89 | 0.151 |
| rs10941679 | A | G | G | 0.25 | 1.13 | 0.27 | 0.122 |
| rs10995190 | G | A | G | 0.84 | 0.86 | 0.86 | 0.151 |
| rs11199914 | C | T | C | 0.68 | 0.95 | 0.68 | 0.051 |
| rs11242675 | T | C | T | 0.61 | 0.94 | 0.64 | 0.062 |
| rs11249433 | A | G | G | 0.40 | 1.09 | 0.41 | 0.086 |
| rs11552449 | C | T | T | 0.17 | 1.07 | 0.17 | 0.068 |
| rs11571833 | A | T | T | 0.01 | 1.26 | 0.01 | 0.231 |
| rs11780156 | C | T | T | 0.16 | 1.07 | 0.19 | 0.068 |
| rs11814448 | A | C | C | 0.02 | 1.26 | 0.03 | 0.231 |
| rs11820646 | C | T | C | 0.59 | 0.95 | 0.60 | 0.051 |
| rs12422552 | G | C | C | 0.26 | 1.05 | 0.27 | 0.049 |
| rs1292011 | A | G | A | 0.58 | 0.92 | 0.59 | 0.083 |
| rs132390 | T | C | C | 0.04 | 1.12 | 0.03 | 0.113 |
| rs13329835 | A | G | G | 0.22 | 1.08 | 0.23 | 0.077 |
| rs13387042 | A | G | A | 0.51 | 0.88 | 0.53 | 0.128 |
| rs1353747 | T | G | T | 0.905 | 0.92 | 0.91 | 0.083 |
| rs1432679 | T | C | C | 0.43 | 1.07 | 0.45 | 0.068 |
| rs1436904 | T | G | T | 0.60 | 0.96 | 0.61 | 0.041 |
| rs1550623 | A | G | A | 0.84 | 0.94 | 0.84 | 0.062 |
| rs16857609 | C | T | T | 0.26 | 1.08 | 0.27 | 0.077 |
| rs17356907 | A | G | A | 0.70 | 0.91 | 0.71 | 0.094 |
| rs17530068 | A | G | G | 0.22 | 1.05 | 0.23 | 0.049 |
| rs17726078 | G | A | G | 0.52 | 0.95 | 0.54 | 0.051 |
| rs17817449 | T | G | T | 0.60 | 0.93 | 0.61 | 0.073 |
| rs204247 | A | G | G | 0.43 | 1.05 | 0.44 | 0.049 |
| rs2046210 | G | A | A | 0.34 | 1.08 | 0.36 | 0.077 |
| rs2236007 | G | A | G | 0.79 | 0.93 | 0.79 | 0.073 |
| rs2380205 | C | T | C | 0.56 | 0.98 | 0.57 | 0.020 |
| rs2588809 | C | T | T | 0.16 | 1.08 | 0.17 | 0.077 |
| rs2823093 | G | A | G | 0.73 | 0.92 | 0.73 | 0.083 |
| rs2943559 | A | G | G | 0.07 | 1.13 | 0.09 | 0.122 |
| rs2981582 | G | A | A | 0.40 | 1.25 | 0.41 | 0.223 |
| rs3757318 | G | A | A | 0.07 | 1.16 | 0.08 | 0.148 |
| rs3760982 | G | A | A | 0.46 | 1.06 | 0.47 | 0.058 |
| rs3803662 | G | A | A | 0.26 | 1.24 | 0.29 | 0.215 |
| rs3903072 | G | T | G | 0.53 | 0.95 | 0.53 | 0.051 |
| rs4808801 | A | G | A | 0.65 | 0.93 | 0.66 | 0.073 |
| rs4973768 | C | T | T | 0.47 | 1.10 | 0.49 | 0.095 |
| rs527616 | G | C | G | 0.62 | 0.95 | 0.63 | 0.051 |
| rs6001930 | T | C | C | 0.11 | 1.12 | 0.10 | 0.113 |
| rs614367 | C | T | T | 0.15 | 1.21 | 0.16 | 0.191 |
| rs616488 | A | G | A | 0.67 | 0.94 | 0.68 | 0.062 |
| rs6504950 | G | A | G | 0.72 | 0.94 | 0.73 | 0.062 |
| rs6762644 | A | G | G | 0.40 | 1.07 | 0.38 | 0.068 |
| rs6828523 | C | A | C | 0.87 | 0.90 | 0.88 | 0.105 |
| rs704010 | C | T | T | 0.38 | 1.08 | 0.38 | 0.077 |
| rs7072776 | G | A | A | 0.29 | 1.07 | 0.30 | 0.068 |
| rs720475 | G | A | G | 0.75 | 0.94 | 0.74 | 0.062 |
| rs8170 | G | A | A | 0.19 | 1.04 | 0.18 | 0.039 |
| rs865686 | T | G | T | 0.62 | 0.89 | 0.64 | 0.117 |
| rs889312 | A | C | C | 0.28 | 1.12 | 0.29 | 0.113 |
| rs941764 | A | G | G | 0.34 | 1.06 | 0.35 | 0.058 |
| rs9693444 | C | A | A | 0.32 | 1.07 | 0.33 | 0.068 |
| rs9790517 | C | T | T | 0.23 | 1.05 | 0.22 | 0.049 |
| rs12493607 | G | C | C | 0.35 | 1.06 | 0.35 | 0.058 |
| rs6472903 | T | G | T | 0.82 | 0.91 | 0.84 | 0.094 |
| rs13281615 | A | G | G | 0.41 | 1.09 | 0.43 | 0.086 |
| rs3817198 | T | C | C | 0.31 | 1.07 | 0.33 | 0.068 |
| rs10759243 | C | A | A | 0.39 | 1.06 | 0.31 | 0.058 |
| rs4849887 | C | T | C | 0.90 | 0.91 | 0.89 | 0.094 |
| rs7918599 | A | G | G | 0.46 | 1.06 | 0.46 | 0.058 |

^1^ From the large meta-analysis of nine GWAS studies. ^8^

^2^ Odds ratio (OR) for minor allele from prior studies, with values less than 1.0 if risk allele is major allele.

^3^ Risk allele frequency in NHS and NHS II nested.
