# Supplementary material for: Addition of a polygenic risk score, mammographic density, and endogenous hormones to existing breast cancer risk prediction models: A nested case–control study
Source: PLoS Med. 2018 Sep 4;15(9):e1002644. doi: 10.1371/journal.pmed.1002644 (PMC6122802; doi:10.1371/journal.pmed.1002644)
Supplement: S2 Table — (DOCX) [file pmed.1002644.s004.docx]

**S2 Table. The number of cases and controls with available biological**

**marker data: Nurses' Health Study and Nurses' Health Study II.**

| **Model** | **Women in the full cohorts with both questionnaire* and genetic data** | **Women in the blood subcohort**  **with measured endogenous**  **hormone data**** | **Women in the blood subcohort with measured percent MD data** |
| --- | --- | --- | --- |
| **Gail** | 4,006 ca / 7,874 co | E1S: 558 ca / 1,259 co  T: 550 ca / 1,201 co  PRL: 1,106 ca / 1,724 co | 1,335 ca / 2,944 co |
| **Rosner-Colditz** | 2,676 ca / 5,484 co | E1S: 366 ca / 805 co  T: 378 ca / 858 co  PRL: 744 ca / 1,218 co | 985 ca / 2,303 co |

*: Information on breast cancer risk factors were obtained from the questionnaire data

**: We only included hormones which have been shown associated with breast cancer based on our previous work in the cohorts. Specifically, E1S (estrone sulfate) and T (testosterone) were included only among postmenopausal women not using HT; PRL (prolactin) was included for all postmenopausal women in both cohorts.
