# Supplementary material for: Addition of a polygenic risk score, mammographic density, and endogenous hormones to existing breast cancer risk prediction models: A nested case–control study
Source: PLoS Med. 2018 Sep 4;15(9):e1002644. doi: 10.1371/journal.pmed.1002644 (PMC6122802; doi:10.1371/journal.pmed.1002644)
Supplement: S3 Table — (DOCX) [file pmed.1002644.s005.docx]

**S3 Table. Baseline characteristics of cases and matched controls in the Nurses' Health Study (1990) and Nurses' Health Study II (1997) for the Rosner-Colditz model**

|  | Cases | Controls |
| --- | --- | --- |
| No. of cases/controls* | 2,676 | 5,484 |
| Nurses' Health Study | 1,851 | 2,841 |
| Nurses' Health Study II | 825 | 2,643 |
| Demographic/lifestyle factors |  |  |
| Mean (SD) |  |  |
| Age at blood draw, yr | 53.0 (8.2) | 51.2 (8.4) |
| <40 | 98 (4%) | 325 (6%) |
| 40-44 | 362 (14%) | 1024 (19%) |
| 45-49 | 673 (25%) | 1597 (29%) |
| 50-54 | 468 (17%) | 793 (14%) |
| 55-59 | 423 (16%) | 640 (12%) |
| 60-64 | 393 (15%) | 655 (12%) |
| 65+ | 259 (10%) | 450 (8%) |
| BMI at blood draw, kg/m^2^ | 25.4 (4.0) | 25.6 (4.7) |
| Age at menarche, yr | 12.4 (1.4) | 12.5 (1.4) |
| Age at first birth, yr | 25.8 (3.5) | 25.6 (3.5) |
| Age at menopause, yr | 51.5 (4.7) | 51.1 (5.6) |
| Physical activity, MET-hrs/wk, Mean (SD) | 16.4 (19.0) | 17.2 (19.8) |
| Alcohol consumption, g/d, Mean (SD) | 5.1 (9.3) | 4.5 (8.4) |
| Percent |  |  |
| Parous | 87 | 86 |
| Previous history of benign breast disease | 50 | 42 |
| Family history of breast cancer | 17 | 11 |
| Postmenopausal status | 56 | 50 |
| Postmenopausal women not using HT | 24 | 24 |
| Postmenopausal women using HT | 32 | 26 |
| Mean |  |  |
| Polygenic risk score† | 0.20 | -0.15 |
| Percent of MD | 34.5 | 29.5 |
| Mean 5-year breast cancer risk score |  |  |
| Rosner-Colditz | 0.019 | 0.014 |
| Hormones |  |  |
| Median (10-90th percentile) |  |  |
| Estrone sulfate(pg/mL) ‡ | 264 (105, 660) | 206 (94, 481) |
| Testosterone (ng/dL) ‡ | 21.2 (12.0, 39.0) | 19.5 (10.0, 36.7) |
| Prolactin (ng/mL) § | 10.7 (5.9, 19.7) | 9.7 (5.55,18.3) |

*Cheek cell data were used for 1,741 women (757ca/984co) in the NHS, and 1,860 women (410ca/1,450co) in the NHS II. Although age and menopausal status were matching factors, the addition of unmatched controls from the excluded in situ cases resulted in imbalance on age and menopausal status.

† Polygenic risk score was created using 67 independent SNPs previously identified in GWAS studies, and were weighted by natural logarithm of their respective effect sizes, followed by standardization (conversion to mean = 0 and SD = 1) among all participants. Negative values means that those women are less likely to develop breast cancer than the average-risk women, based on genetic data from these SNPs. Results presented in this table were among women who contributed to Gail model analyses.

‡ Among postmenopausal women not using postmenopausal hormone therapy (HT) (653 cases/1,336 controls).

§ Among postmenopausal women (1,512 cases/2,743 controls)
