# Supplementary material for: Addition of a polygenic risk score, mammographic density, and endogenous hormones to existing breast cancer risk prediction models: A nested case–control study
Source: PLoS Med. 2018 Sep 4;15(9):e1002644. doi: 10.1371/journal.pmed.1002644 (PMC6122802; doi:10.1371/journal.pmed.1002644)
Supplement: S4 Table — (DOCX) [file pmed.1002644.s006.docx]

**S4 Table. Multivariable* relative risk (MV RR) of invasive breast cancer in relation to PRS and MD in the Gail model.**

|  | MV RR* (95% CI) across quartile categories | | | |
| --- | --- | --- | --- | --- |
|  | 1 | 2 | 3 | 4 |
| PRS (4,006/7,874) | 1 (ref) | 1.3 | 1.7 | 2.5 (2.2-2.8) † |
| MD measured (1,335/2,944) | 1 (ref) | 1.3 | 2.0 | 2.8 (2.3-3.5) |
| MD imputed (2,671/4,930) | 1 (ref) | 1.3 | 1.6 | 2.2 (1.9-2.6) |
| MD combined (4,006/7,874) | 1 (ref) | 1.3 | 1.8 | 2.4 (2.2-2.8) ‡ |

* We adjusted for age at blood draw (continuous), BMI at blood draw (<25, 25-<30, ≥30 kg/m^2^), fasting status (<8 hours, ≥8 hours), time of day (24 hour clock: <8, 8-12, 13-24) and season of blood draw (May to October, other months), history of benign breast disease (no, yes), family history of breast cancer (no, yes), age at menopause (continuous), age at menarche (<12,12,13, or ≥14 y), physical activity (<3, 3-27,>27 MET-hrs/wk), and age at first birth and parity (nulliparous; 1-4 children, first birth<25 y; 1-4 children, first birth 25-29 y; 1-4 children, first birth ≥30 y; ≥5 children, first birth<25 y; or ≥5children, first birth ≥25 y).

† The MV RR was 2.4 (2.2-2.7) for PRS adjusted for MD;

‡ The MV RR was 2.4 (2.1-2.7) for MD adjusted for PRS.
