# Supplementary material for: Addition of a polygenic risk score, mammographic density, and endogenous hormones to existing breast cancer risk prediction models: A nested case–control study
Source: PLoS Med. 2018 Sep 4;15(9):e1002644. doi: 10.1371/journal.pmed.1002644 (PMC6122802; doi:10.1371/journal.pmed.1002644)
Supplement: S5 Table — (DOCX) [file pmed.1002644.s007.docx]

**S5 Table. Multivariable* relative risk (MV RR) of invasive breast cancer in relation to circulating pre-diagnostic hormones in the Gail model**

|  | MV RR* (95% CI) across quartile categories | | | |
| --- | --- | --- | --- | --- |
|  | 1 | 2 | 3 | 4** |
| **T (Testosterone)†** |  |  |  |  |
| Measured (550/1,201) | 1 (ref) | 0.9 | 1.3 | 1.5 (1.1-2.0) |
| First imputation (1,005/2,070) ‡ | 1 (ref) | 1.2 | 1.4 | 1.7 (1.4-2.1) |
| Average of 5 imputations (1,005/2,070) | 1 (ref) | 1.0 | 1.4 | 1.7 (1.3-2.1) |
| **E1S (Estrone sulfate)†** |  |  |  |  |
| Measured (558/1,259) | 1 (ref) | 1.1 | 1.4 | 2.0 (1.4-2.6) |
| First imputation (977/1,937) ‡ | 1 (ref) | 1.3 | 1.7 | 2.3 (1.8-2.9) |
| Average of 5 imputations (977/1,937) | 1 (ref) | 1.2 | 1.6 | 2.1 (1.5-2.8) |
| **PRL (Prolactin)** § |  |  |  |  |
| Measured (1,106/1,724) | 1 (ref) | 0.9 | 1.3 | 1.4 (1.2-1.8) |
| First imputation (2,435/4,349) ‡ | 1 (ref) | 1.0 | 1.3 | 1.4 (1.2-1.6) |
| Average of 5 imputations (2,435/4,349) | 1 (ref) | 1.0 | 1.4 | 1.4 (1.2-1.7) |

* We adjusted for age at blood draw (continuous), BMI at blood draw (<25, 25-<30, ≥30 kg/m^2^), fasting status (<8 hours, ≥8 hours), time of day (24 hour clock: <8, 8-12, 13-24), season of blood draw (May to October, other months), history of benign breast disease (no, yes), family history of breast cancer (no, yes), age at menopause (continuous), age at menarche (<12,12,13, or ≥14 y), physical activity (<3, 3-27, >27 MET-hrs/wk), and age at first birth and parity (nulliparous; 1-4 children, first birth<25 y; 1-4 children, first birth 25-29 y; (1-4 children, first birth ≥30 y; ≥5 children, first birth<25 y; or ≥5children, first birth ≥25 y).

** After further adjustment for PRS and MD, the MV RRs (95%CIs) in this quartile category for the measured data and first imputed data were 1.6 (1.1-2.5) and 1.7 (1.1-2.5) for T; 1.9 (1.2-2.9) and 2.1 (1.4-3.3) for E1S; and 1.6 (1.2-2.2) and 1.3 (1.0-1.7) for PRL.

† Among postmenopausal women not using HT.

‡ Using the measured and first imputed data.

§ Among all postmenopausal women.
