# Supplementary material for: Addition of a polygenic risk score, mammographic density, and endogenous hormones to existing breast cancer risk prediction models: A nested case–control study
Source: PLoS Med. 2018 Sep 4;15(9):e1002644. doi: 10.1371/journal.pmed.1002644 (PMC6122802; doi:10.1371/journal.pmed.1002644)
Supplement: S6 Table — (DOCX) [file pmed.1002644.s008.docx]

**S6 Table. Change in age-adjusted AUC of Gail model by age group for invasive breast cancer by including PRS, MD, and circulating hormones**

|  | **No. ca/co** | **Baseline AUC* (95%CI)** | **Full model** AUC (95%CI)** | **Changes in AUC (95%CI)** |
| --- | --- | --- | --- | --- |
| **Premenopausal women** |  |  |  |  |
| <40 | 137/412 | 51.0 (45.5-56.5) | 65.1(59.8-70.4) | 14.2 (6.9-21.5) |
| 40-44 | 374/1,037 | 57.4 (54.1-60.7) | 62.6 (59.3-65.9) | 5.2 (0.9-9.5) |
| 45-49 | 677/1,457 | 56.0 (53.5-58.5) | 65.4 (62.9-67.9) | 9.4 (6.3-12.5) |
| 50-54 | 328/553 | 57.0 (53.1-60.9) | 62.9 (59.2-66.6) | 5.9 (1.4-10.4) |
| 55-59 | 55/66 | 49.3 (38.9-59.7) | 62.2 (52.2-72.2) | 12.9 (1.1-24.7) |
|  | 1,571/3,525† | 55.9 (54.1-57.7) | 64.1 (62.5-65.7) | 8.2 (6.0-10.4) |
| **Postmenopausal women not using HT** | |  |  |  |
| <45 | 14/41 | 65.8 (49.3-82.3) | 69.7 (53.8-85.6) | 3.9 (-17.7-25.5) |
| 45-49 | 71/155 | 54.3 (46.3-62.3) | 64.6 (57.0-72.2) | 10.3 (0.1-20.5) |
| 50-54 | 149/306 | 58.4 (52.9-63.9) | 67.6 (62.3-72.9) | 9.2 (2.5-15.9) |
| 55-59 | 266/539 | 56.2 (52.1-60.3) | 67.7 (63.8-71.6) | 11.6 (6.5-16.7) |
| 60-64 | 299/604 | 53.6 (49.7-57.5) | 62.7 (58.8-66.6) | 9.1 (4.4-13.8) |
| 65-69 | 206/425 | 54.5 (49.8-59.2) | 67.1 (62.6-71.6) | 12.5 (6.8-18.2) |
|  | 1,005/2,070† | 55.5 (53.3-57.7) | 66.0 (64.0-68.0) | 10.5 (8.0-13.0) |
| **Postmenopausal women using HT** |  |  |  |  |
| <45 | 29/112 | 60.1 (48.5-71.7) | 69.0 (58.2-79.8) | 8.9 (-5.2-23.0) |
| 45-49 | 180/444 | 60.0 (55.1-64.9) | 67.4 (62.7-72.1) | 7.4 (1.3-13.5) |
| 50-54 | 292/536 | 61.8 (57.9-65.7) | 65.8 (61.9-69.7) | 4.0 (-0.9-8.9) |
| 55-59 | 390/501 | 56.3 (52.6-60.0) | 64.1 (60.4-67.8) | 7.8 (3.3-12.3) |
| 60-64 | 336/412 | 54.6 (50.5-58.7) | 65.1 (61.2-69.0) | 10.5 (6.0-15.0) |
| 65-69 | 202/274 | 57.7 (52.6-62.8) | 60.5 (55.4-65.6) | 2.8 (-3.1-8.7) |
| 70-74 | 1/0 | -- | -- | -- |
|  | 1,430/2,279† | 58.0 (56.0-60.0) | 64.9 (63.1-66.7) | 6.9 (4.7-9.1) |
| **All women** |  |  |  |  |
| <40 | 139/434 | 51.4 (45.9-56.9) | 65.5 (60.4-70.6) | 14.1 (6.8-21.4) |
| 40-44 | 415/1,168 | 57.6 (54.5-60.7) | 62.7 (59.6-65.8) | 5.1 (0.8-9.4) |
| 45-49 | 928/2,056 | 56.7 (54.5-58.9) | 65.2 (63.0-67.4) | 8.5 (5.8-11.2) |
| 50-54 | 769/1,395 | 58.9 (56.4-61.4) | 64.5 (62.1-66.9) | 5.6 (2.5-8.7) |
| 55-59 | 711/1,106 | 55.2 (52.5-57.9) | 66.4 (63.9-68.9) | 11.2 (7.9-14.5) |
| 60-64 | 635/1,016 | 53.8 (50.9-56.7) | 65.1 (62.4-67.8) | 11.3 (8.0-14.6) |
| 65-69 | 408/699 | 55.9 (52.4-59.4) | 65.3 (62.0-68.6) | 9.4 (5.3-13.5) |
| 70-74 | 1/0 | -- | -- | -- |
|  | 4,006/7,874† | 56.3 (55.1-57.5) | 65.0 (64.0-66.0) | 8.8 (7.4-10.2) |

*These are “baseline” AUC of the Gail model without any of the biomarkers included. Some of the changes in AUC did not match exactly due to rounding.

**For the full model, postmenopausal women, we included PRS and MD for the premenopausal women, PRS, MD, T, E1S, PRL for the postmenopausal women not using HT, and PRS, MD and PRL for the postmenopausal women using HT. PRS, MD, and circulating hormones were modeled as continuous variables.

†: We conducted meta-analyses of the age-adjusted AUCs to remove the effect of age.
