# Supplementary material for: Addition of a polygenic risk score, mammographic density, and endogenous hormones to existing breast cancer risk prediction models: A nested case–control study
Source: PLoS Med. 2018 Sep 4;15(9):e1002644. doi: 10.1371/journal.pmed.1002644 (PMC6122802; doi:10.1371/journal.pmed.1002644)
Supplement: S9 Table — (DOCX) [file pmed.1002644.s011.docx]

**S9 Table. Change in age-adjusted AUC of Rosner-Colditz model for invasive breast cancer by including PRS, measured MD, and measured circulating hormones**

|  | **No. ca/co** | **Baseline**  **AUC (95%CI)*** | **Full model**  **AUC (95%CI)** | **Change in AUC (95%CI)** |
| --- | --- | --- | --- | --- |
| **Premenopausal women** |  |  |  |  |
| **+ MD** | 467/1,218 | 62.2 (59.3-65.1) | 64.5 (61.6-67.4) | 2.3 (0.3-4.3) |
| **+ PRS + MD** | 467/1,218 | 62.2 (59.3-65.1) | 69.5 (66.8-72.2) | 7.4 (4.7-10.1) |
| **Postmenopausal women not using HT** | |  |  |  |
| **+ MD** | 229/567 | 58.2 (53.9-62.5) | 58.6 (54.3-62.9) | 0.5 (-1.5-2.5) |
| **+ T + E1S + PRL** | 287/622 | 60.2 (56.3-64.1) | 63.7 (59.8-67.6) | 3.7 (1.0-6.4) |
| **+ PRS + MD** | 229/567 | 58.2 (53.9-62.5) | 64.0 (59.9-68.1) | 5.8 (1.9-9.7) |
| **+ PRS + T + E1S + PRL** | 287/622 | 60.2 (56.3-64.1) | 65.1 (61.4-68.8) | 5.1 (1.8-8.4) |
| **+ MD + T + E1S + PRL** | 182/419 | 58.8 (53.9-63.7) | 64.5 (59.8-69.2) | 5.9 (2.0-9.8) |
| **+ PRS + MD + T + E1S + PRL**† | 182/419 | 58.7 (53.8-63.6) | 67.3 (62.6-72.0) | 8.6 (3.9-13.3) |
| **Postmenopausal women using HT** |  |  |  |  |
| **+ MD** | 289/518 | 60.1 (56.0-64.2) | 64.0 (60.1-67.9) | 3.7 (0.4-7.0) |
| **+ PRL** | 437/543 | 59.7 (56.2-63.2) | 60.6 (57.1-64.1) | 0.8 (-0.4-2.0) |
| **+ PRS + MD** | 289/518 | 60.1 (56.0-64.2) | 68.3 (64.4-72.2) | 7.8 (3.9-11.7) |
| **+ PRS + PRL** | 255/358 | 57.8 (53.3-62.3) | 65.0 (60.7-69.3) | 7.0 (2.9-11.1) |
| **+ MD + PRL** | 255/358 | 57.8 (53.3-62.3) | 61.7 (57.2-66.2) | 3.9 (0.2-7.6) |
| **+ PRS + MD + prolactin** | 255/358 | 57.8 (53.3-62.3) | 66.3 (62.0-70.6) | 8.5 (4.2-12.8) |
| **All women** | 786/1,561 | 61.6 (59.2-64.0) | 68.2 (65.8-70.6) | 6.6 (4.4-8.8) |

*These are “baseline” AUC without any of the biomarkers included. For the full model AUC, PRS, MD, and circulating hormones were modeled as continuous variables. Some of the changes in AUC did not match exactly due to rounding.

† Because of the few number of cases and control if using <45 as the first age group. We used <49 (rather than <45) as the first age group when we conducted the age-stratified analysis for this subgroup.
