# Supplementary material for: Addition of a polygenic risk score, mammographic density, and endogenous hormones to existing breast cancer risk prediction models: A nested case–control study
Source: PLoS Med. 2018 Sep 4;15(9):e1002644. doi: 10.1371/journal.pmed.1002644 (PMC6122802; doi:10.1371/journal.pmed.1002644)
Supplement: S10 Table — (DOCX) [file pmed.1002644.s012.docx]

**S10 Table. Change in age-adjusted AUC of Gail and Rosner-Colditz model for ER+ breast cancer by including PRS, MD, and circulating hormones among postmenopausal women not using HT**

|  | **No. ca/co** | **AUC (95%CI)** | **Changes in AUC (95%CI)** |
| --- | --- | --- | --- |
| **Gail model** | 772/2,070 | 53.1 (50.7-55.5)* |  |
| **+ PRS** |  | 62.0 (59.6-64.4) | 9.0 (6.1-11.9) |
| **+ MD** |  | 57.5 (55.1-59.9) | 4.4 (1.9-6.9) |
| **+ T + E1S + PRL** |  | 62.8 (60.4-65.2) | 9.7 (6.8-12.6) |
| **+ PRS + MD** |  | 63.3 (60.9-65.7) | 10.4 (7.5-13.3) |
| **+ PRS + T + E1S + PRL** |  | 66.0 (63.8-68.2) | 13.0 (10.1-15.9) |
| **+ MD + T + E1S + PRL** |  | 64.4 (62.0-66.8) | 11.4 (8.5-14.3) |
| **+ PRS + MD + T + E1S + PRL** |  | 67.3 (65.1-69.5) | 14.3 (11.4-17.2) |
| **Rosner-Colditz model** | 520/1,336 | 60.8 (58.1-63.5)* |  |
| **+ PRS** |  | 65.5 (62.8-68.2) | 4.9 (2.5-7.3) |
| **+ MD** |  | 60.9 (58.2-63.6) | 0.7 (-0.7-2.1) |
| **+ T + E1S + PRL** |  | 64.5 (61.8-67.2) | 3.8 (1.8-5.8) |
| **+ PRS + MD** |  | 65.7 (63.0-68.4) | 5.2 (2.7-7.7) |
| **+ PRS + T + E1S + PRL** |  | 67.4 (64.7-70.1) | 6.9 (4.4-9.4) |
| **+ MD + T + E1S + PRL** |  | 64.9 (62.2-67.6) | 4.4 (2.2-6.6) |
| **+ PRS + MD + T + E1S + PRL** |  | 67.8 (65.1-70.5) | 7.3 (4.8-9.8) |

*These are “baseline” AUC without any of the biomarkers included. PRS, MD, and circulating hormones were modeled as continuous variables. Some of the changes in AUC did not match exactly due to rounding.
