# Supplementary material for: Addition of a polygenic risk score, mammographic density, and endogenous hormones to existing breast cancer risk prediction models: A nested case–control study
Source: PLoS Med. 2018 Sep 4;15(9):e1002644. doi: 10.1371/journal.pmed.1002644 (PMC6122802; doi:10.1371/journal.pmed.1002644)
Supplement: S11 Table — (DOCX) [file pmed.1002644.s013.docx]

**S11 Table. Beta coefficients for each of the input parameters used in Tables 2 and 3.**

|  | Intercept | Risk score | PRS | MD | T | E1S | PRL |
| --- | --- | --- | --- | --- | --- | --- | --- |
| **Gail model** |  |  |  |  |  |  |  |
| Premenopausal women | -3.2472 | 0.2455 | 0.3415 | 0.0169 | NA | NA | NA |
| Postmenopausal women not using HT | -2.5087 | 0.0987 | 0.3269 | 0.015 | 0.0133 | 0.00119 | 0.00888 |
| Postmenopausal women using HT | -3.4733 | 0.1561 | 0.3468 | 0.0198 | NA | NA | 0.0122 |
| **Rosner-Colditz model** | |  |  |  |  |  |  |
| Premenopausal women | -2.3247 | 0.4927 | 0.3423 | 0.0152 | NA | NA | NA |
| Postmenopausal women not using HT | -2.1345 | 0.3818 | 0.3328 | 0.0126 | 0.018 | 0.00113 | 0.00787 |
| Postmenopausal women using HT | -3.5692 | 0.2975 | 0.4475 | 0.0156 | NA | NA | 0.0203 |
